# Development and external validation of a nomogram to predict overall survival following stereotactic body radiotherapy for early-stage lung cancer

**DOI:** 10.1186/s13014-020-01537-z

**Published:** 2020-04-22

**Authors:** Sarah Baker, Katerina Bakunina, Marloes Duijm, Mischa S. Hoogeman, Robin Cornelissen, Imogeen Antonisse, John Praag, Wilma D. Heemsbergen, Joost Jan Nuyttens

**Affiliations:** 1 Department of Radiation Oncology, Erasmus MC Cancer Institute, Groene Hilledijk 302, Postbus 2040, 3000 CA Rotterdam, The Netherlands; 2 Department of Pulmonary Medicine, Erasmus MC Cancer Institute, s Gravendijkwal 230, 3015 CD Rotterdam, The Netherlands

**Keywords:** Non-small cell, Lung neoplasm, Radiation, Individual survival prediction

## Abstract

**Background:**

Prognostication tools for early-stage non-small cell lung cancer (NSCLC) patients treated with stereotactic body radiotherapy (SBRT) are currently lacking. The purpose of this study was to develop and externally validate a nomogram to predict overall survival in individual patients with peripheral early-stage disease.

**Methods:**

A total of 587 NSCLC patients treated with biologically effective dose > 100 Gy_10_ were eligible. A Cox proportional hazards model was used to build a nomogram to predict 6-month, 1-year, 3-year and 5-year overall survival. Internal validation was performed using bootstrap sampling. External validation was performed in a separate cohort of 124 NSCLC patients with central tumors treated with SBRT. Discriminatory ability was measured by the concordance index (C-index) while predictive accuracy was assessed with calibration slope and plots.

**Results:**

The resulting nomogram was based on six prognostic factors: age, sex, Karnofsky Performance Status, operability, Charlson Comorbidity Index, and tumor diameter. The slope of the calibration curve for nomogram-predicted versus Kaplan-Meier-estimated overall survival was 0.77. The C-index of the nomogram (corrected for optimism) was moderate at 0.64. In the external validation cohort, the model yielded a C-index of 0.62.

**Conclusions:**

We established and validated a nomogram which can provide individual survival predictions for patients with early stage lung cancer treated with SBRT. The nomogram may assist patients and clinicians with treatment decision-making.

## Background

Stereotactic body radiotherapy (SBRT) is the standard of care for medically inoperable early-stage non-small cell lung cancer (NSCLC) [1]. It is increasingly utilized also in the high risk operable patient population [2]. Survival outcomes, however, are variable, and predicting survival in this patient population has proven challenging [3, 4].

A major contributor to survival variability is the potentially high rate of competing non-cancer mortality. For example, severe chronic obstructive pulmonary disease (COPD), common in the SBRT lung population, is associated with a 70% mortality rate at 5 years in those with 3 or more acute exacerbations [5]. The proven safety of SBRT in elderly patients [6] and those with severe COPD [7, 8] has promoted an inclusive stance to patient eligibility. Consequently, despite high rates of local control and cancer-specific survival, overall survival (OS) remains poor and in the order of 40% at 5 years [2].

There is currently a paucity of accurate prognostic models for the early lung SBRT population. One study in the United Stated suggested the decision between curative-intent treatment and observation may be driven largely by institutional factors (academic vs non-academic) and patient financial or racial disparities rather than clinical factors or prognosis [9]. The ability to accurately predict survival on the individual patient level would be highly valuable. Not only would it assist patients with future planning and facilitate shared decision-making with clinicians, but it would also allow for judicious resource-allocation and potentially identify patients better served by a supportive care approach. Finally, it would allow for more accurate risk-stratification for clinical trials and comparative outcomes research.

Nomograms are a practical tool which incorporate prognostic factors for a given patient to calculate the expected probability of a clinical event such as 5-year overall survival. In resected early-stage NSCLC [10] as well as in a diverse lung cancer population undergoing a variety of treatments [11], nomograms have proven more accurate than TNM staging for survival prediction. The purpose of this study was to identify prognostic factors for survival in early lung cancer patients treated with SBRT and to build a nomogram to predict 6-month, 1-year, 3-year and 5-year overall survival.

## Methods

### Patients and treatment

Consecutive NSCLC patients treated between August 2005 and January 2017 with 4-dimensional SBRT at Erasmus MC were identified. Patients lacking histologic confirmation had findings on positron emission tomography (PET)-CT scan consistent with early-stage NSCLC, and had been recommended for SBRT by a multidisciplinary tumor board. Treatment planning protocols and follow-up schedule have been previously described [12, 13]. Inclusion criteria included peripheral early-stage disease (T1-T3 N0M0). Exclusion criteria included central location (within 2 cm of the proximal bronchial tree), synchronous intrapulmonary lesions, a diagnosis of small cell lung cancer, and delivered biologically effective dose (BED) < 100 Gy assuming an α/β ratio of 10. Tumor staging was originally performed according to AJCC 7th edition based on PET-CT scan (all patients received a staging PET scan) and patients were re-staged by AJCC 8th edition criteria for the present study. Mediastinal staging was performed by PET-CT, mediastinoscopy, and/or endoscopic ultrasound (EBUS and/or EUS).

### Endpoints and covariates

The primary endpoint was OS at 5 years, calculated from first day of treatment until death, and patients still alive were censored at the date of last follow-up visit. Variables analyzed for association with survival included age, sex, Karnofsky Performance Status (KPS), operability, Charlson Comorbidity Index score (CCI), Cumulative Illness Rating Score (CIRS), smoking status (current/former vs never), Global Initiative for Chronic Obstructive Lung Disease (GOLD) score [14], previous malignancy, previous lung cancer, maximum axial tumor diameter, histology, and lower lobe location. GOLD scores were not reclassified to reflect 2017 criteria, which incorporate a comprehensive assessment of symptoms by validated questionnaires [15], as this data was not available retrospectively. Operability was determined by criteria as outlined in recently published clinical practice guidelines by the European Society for Medical Oncology (based primary on cardiac assessment and pulmonary function) [16].

### Statistical analysis

#### Model building

The nomogram was based on a Cox proportional hazards model, using the following step-wise model building procedure. Variables with more than 1% missing values (histology 57% and smoking status 11%) were omitted from the initial model, and the decision regarding imputation (and then inclusion in the model) made subsequently based on assessment of their potential added predictive value with Cox univariate and multivariate analyses. Complete case analysis was used for variables with less than 1% missing values. The data provided evidence for interaction between the variables GOLD score, age and sex (*p* = 0.001) with the results difficult to interpret and depict in a nomogram (see further description within Results: Nomogram), and therefore GOLD score was not initially included. Thus, an initial model was built using the prognostic factors sex, age, KPS, operability, previous malignancy, previous lung cancer, lower lobe tumor location, and tumor diameter.

The model building steps were formulated as strict programmable decision rules aimed at arriving at the most parsimonious model with maximum predictive ability, so that the model building procedure could be internally validated. Initially, the prognostic factors were modeled flexibly, e.g. allowing highly non-linear relationships. Subsequently, following a predefined grid, less flexible functions were applied. The simplification was thus stopped once it began to come at the price of compromised model fit as compared to the most flexible model. Depending on the distribution of the prognostic factor, suitable measures of fit were used. Age and tumor diameter were modelled flexibly using restricted cubic splines (RCS) with 5 degrees of freedom (d.f.), and KPS was modelled as a nominal variable to allow maximum flexibility. To this model, in turn, RCS functions of CCI and CIRS of 4 d.f. each were added. Goodness-of-fit of each of these models was evaluated with respect to the initial model using a likelihood ratio test (LRT). The comorbidity index (CCI or CIRS) which resulted in a smaller *p*-value was selected (and the resulting model referred to as the full model henceforth). Subsequently, a gradient of RCS functions with d.f. ranging between 2 and 4 and a linear function of the comorbidity score were compared with a LRT to the full model. The functional form with the smallest *p*-value was selected. The effect of age and tumor diameter were modelled simultaneously and evaluated using Akaike’s Information Criteria (AIC) as the compared models are not nested, as suggested by Harrel [17]. The range of RCS of 5 d.f. to linear effect was evaluated. The model with the smallest AIC was selected. The variables previous malignancy and previous lung cancer were also assessed simultaneously using LRT with a *p*-value cut-off point of 0.1 for inclusion in the model. Sex, lower lobe tumor location, and operability were evaluated independently against a threshold for model inclusion of *p* = 0.1 from a LRT, a cut-off value chosen so that the model building procedure could be automated and then validated. Alternative functional forms of KPS score were also evaluated (linear and RCS with 2 and 3 d.f.) and compared to nominal variable modelling using AIC. The model with the smallest AIC is the final model.

#### Internal validation of the model building procedure

The model building procedure was validated by applying it to 1000 bootstrap samples and predicting the original sample based on the resulting model. Discriminative ability of the model was measured with the concordance index (C-index). Internal validation was also used to assess the degree of overfitting to the sample at hand (calibration slope), and the resulting optimism in C-index. The estimated optimism-corrected calibration slope was then used to shrink model predictions and thus increase their external validity [17]. Calibration plots in 1000 bootstrap samples were used to compare Kaplan-Meier-estimated and nomogram-predicted 6-month, 1-year, 3-year and 5-year OS.

#### External validation

An independent cohort of 124 NSCLC patients with centrally located tumors treated with SBRT at Erasmus MC between September 2004 – November 2016 was used for external validation.

The final model underlying the nomogram was used to predict 6-month, 1-year, 3-year, and 5-year OS of the patients in the external validation cohort. The model’s discriminative ability in this cohort was measured using the C-index. For the construction of the calibration plots, the predicted survival probabilities were grouped in four equally sized groups.

Statistical analyses were performed using IBM SPSS statistics version 22.0 software package (SPSS Inc., Chicago, IL, USA) and R software, version 3.4.1 (open source; www.r-project.org).

The study protocol was approved by the Medical Ethical Committee of the Erasmus Medical Center (MEC201679).

## Results

### Patients

A total of 587 patients met inclusion criteria. Baseline clinical and treatment characteristics are shown in Table 1. Median age was 75 years (range 44–91) with median CCI of 3 (range 0–10). Two-hundred and fifty-eight patients had biopsy confirmation of disease, while the remaining 329 had an FDG-avid lesion on PET deemed highly suspicious of NSCLC upon multidisciplinary tumor board review. Mediastinal staging was by PET for the majority of patients (*n* = 478) and invasive staging (mediastinoscopy, EBUS or EUS) was performed in 109 patients.

**Table 1:** Baseline clinical and treatment characteristics of the primary cohort and validation cohort

Variable	Primary cohort		Validation cohort	
	Total N	N or Median (% or range)	Total N	N or Median (% or range)
**Age**	587	75 (44–91)	124	77 (48–90)
**Sex**	587		124	
Female		224 (38%)		46 (37.1%)
Male		363 (62%)		78 (62.9%)
**KPS**	581		124	
≥ 90		117 (20%)		54 (43.5%)
70–80		395 (68%)		62 (50.0%)
≤ 60		69 (12%)		8 (6.5%)
**Operable**	581	40 (7%)	124	10 (8.1%)
**CCI**	587	3 (0–10)	124	2 (0–9)
**CIRS**	587	5 (0–15)	124	5 (0–16)
**Current/former smoker**	521	481 (92%)		
**GOLD score**	580			
1		97 (17%)		
2		240 (41%)		
3		144 (25%)		
4		37 (6%)		
**Previous malignancy**	587	237 (40%)		
**Previous lung cancer**	587	120 (20.4%)		
**T stage**	587		124	
T1		412 (70%)		13 (10.5%)
T2		147 (25%)		55 (44.4%)
T3		28 (5%)		42 (33.9%)
T4		0 (0%)		14 (11.3%)
**Tumor diameter**	587	2.3 cm (0.7–7.7)	124	4.6 cm (1.4–10.5)
**Pathology**	587		124	
Unknown		329 (56%)		
Squamous cell carcinoma		94 (16%)		
Adenocarcinoma		103 (18%)		
Large cell carcinoma		51 (9%)		
Other		10 (2%)		
**Dose fractionation**	587		124	
60 Gy/3		209 (36%)		
54 Gy/3		15 (3%)		
51 Gy/3		354 (60%)		
40 Gy/2		1 (0.2%)		
60 Gy/5		8 (1%)		
55 Gy/5				48 (38.4%)
48 Gy/6				19 (15.2%)
49 Gy/7				17 (13.7%)
60 Gy/5				18 (14.4%)
Other				22 (17.6%)

Abbreviations: *CCI* Charlson Comorbidity Index score, *CIRS* Cumulative Illness Rating Score, *GOLD* Global Initiative for Chronic Obstructive Lung Disease, *KPS* Karnofsky Performance Status

The external validation set consisted of 124 NSCLC patients with centrally located tumors treated with SBRT to a median dose of 55 Gy in 5 fractions. Baseline patient and tumor characteristics were similar to those of the primary patient cohort, however, median tumor diameter was larger and several patients had T4 tumors in the validation cohort (Table 1).

### Survival

**Fig. 1:**
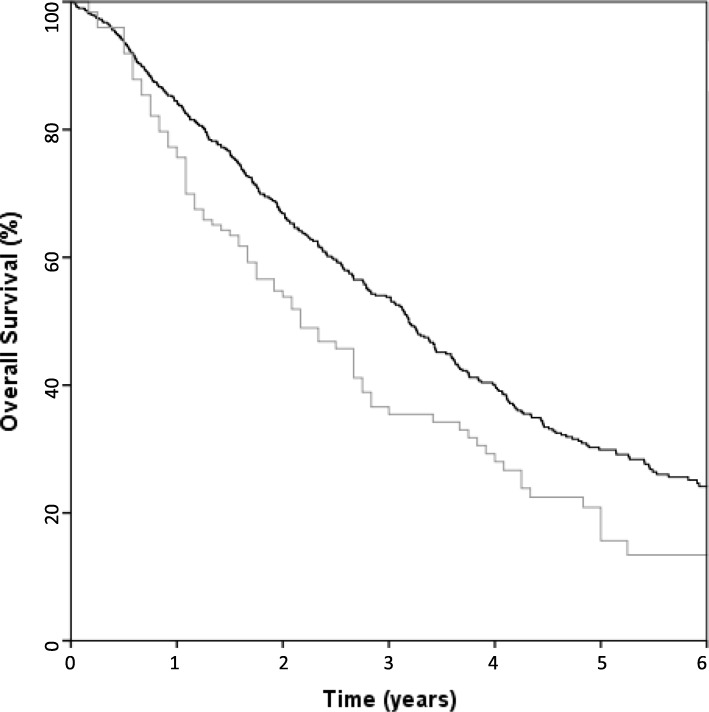
Kaplan-Meier curves showing the overall survival of the original cohort (black line) and validation cohort (grey line)

At the time of analysis, 252 patients (42.9%) were alive. Median follow-up time was 23.8 months (range 0.3–124.6) for all patients and 28.5 months (range 4.5–124.6) for surviving patients. Median OS was 38.4 months (95% confidence interval [CI] 34.2–42.6). Three-year and 5-year OS were 54.2 and 29.9%, respectively (Fig. 1). Median follow-up time in the validation cohort was 22.3 months (range 1.9–121.2) and median OS was 26.0 months (95% CI 19.5–32.5) (Fig. 1).

### Nomogram

**Fig. 2:**
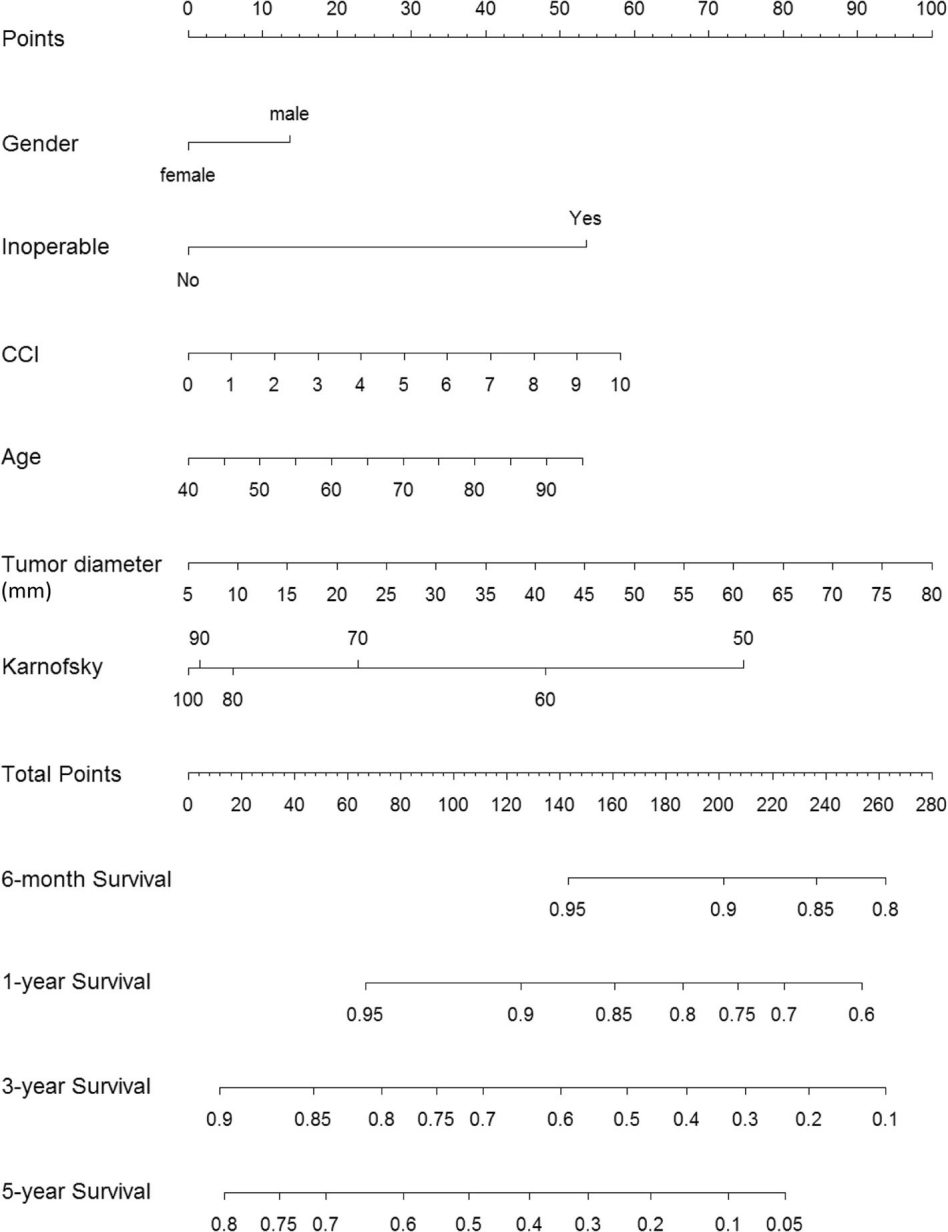
Nomogram for prediction of 6-month, 1-year, 3-year and 5-year survival. Abbreviations: CCI Charlson Comorbidity Index score

**Fig. 3:**
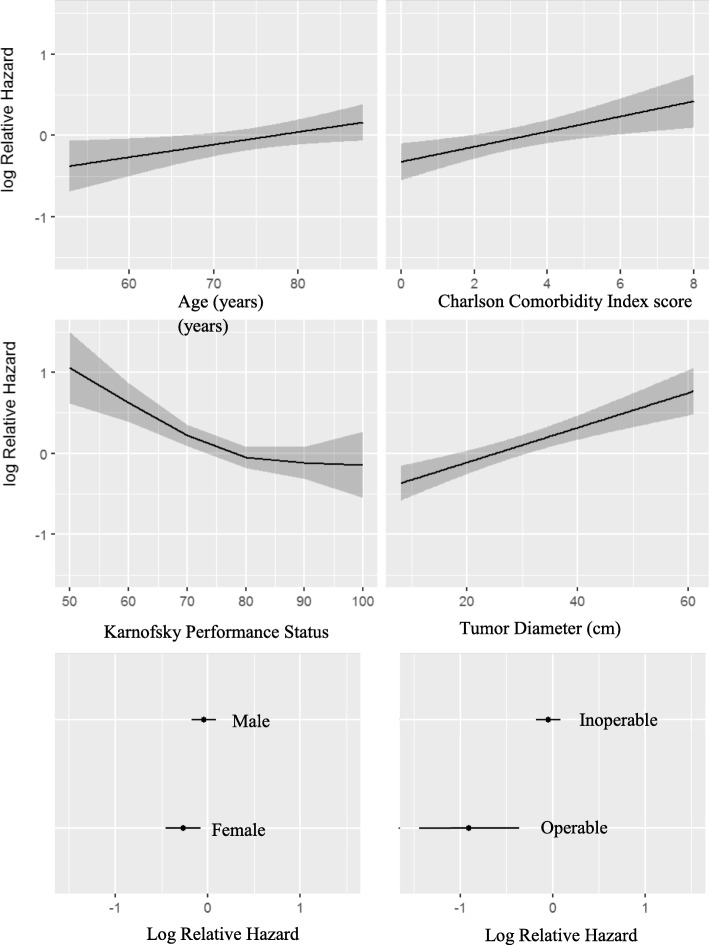
Relative hazard of death modelled for each variable included in the nomogram. The gray areas (first 4 panels) and the horizontal black bars (last 2 panels) depict the 95% confidence intervals

Six patients with unknown KPS score were omitted from the nomogram building procedure. Application of the model building procedure to the remaining 581 patients resulted in a final model based on the variables age, sex, operability, KPS, CCI and tumor diameter. The resulting nomogram is presented in Fig. 2. While age, CCI and tumor diameter were modelled as linear functions, KPS was best modelled as a quadratic function with restriction to linearity at both extremes of the scale, i.e. RCS function with 2 d.f. (Fig. 3). Univariate analysis demonstrated no additional predictive value from including histology, smoking status or GOLD score (*p*-values 0.38, 0.39, and 0.16, respectively). When added to the final model, histology and smoking remained insignificant and thus these variables were not included in the model. Conversely, GOLD score proved significant (p-value 0.004) when modeled as a nominal variable, however, survival effects were paradoxical: with respect to GOLD 0, GOLD 4 had a nearly identical effect on OS (HR 1.01 *p* = 0.76) while GOLD 1–3 showed favorable effects on survival with respect to GOLD 0 (HR 0.68, 0.57 and 0.63, and *p*-values 0.066, 0.002, and 0.022, respectively). When trying to understand these findings, we performed interaction tests with age and sex, which were significant (Chi2 43.7, 19 d.f., *p* = 0.001). In order to preserve greater parsimony and nomogram readability, and given the paradoxical effect of GOLD score severity on survival, GOLD score was not included in the model. The parameter estimates of the final model are shown in Table 2.

**Table 2:** Parameter estimates of the final model used to generate the nomogram

	HR	SE	*p*-value
Sex (male vs female)	1.245	0.124	0.079
Inoperable (yes vs No)	2.361	0.285	0.003
CCI^a^	1.098	0.031	0.002
Age^a^	1.016	0.007	0.023
Tumor diameter^a^	1.022	0.004	< 0.001
KPS^a,b^ (linear effect)	0.958	0.011	< 0.001
KPS^a,b^ (quadratic effect)	1.020	0.011	0.057

^a^ Per unit increase

^b^ Restricted cubic splines function parameters

Abbreviations: *CCI* Charlson Comorbidity Index score, *KPS* Karnofsky performance status, *HR* hazard ratio, *SE* standard error of the log-hazard ratio

### Validation

**Fig. 4:**
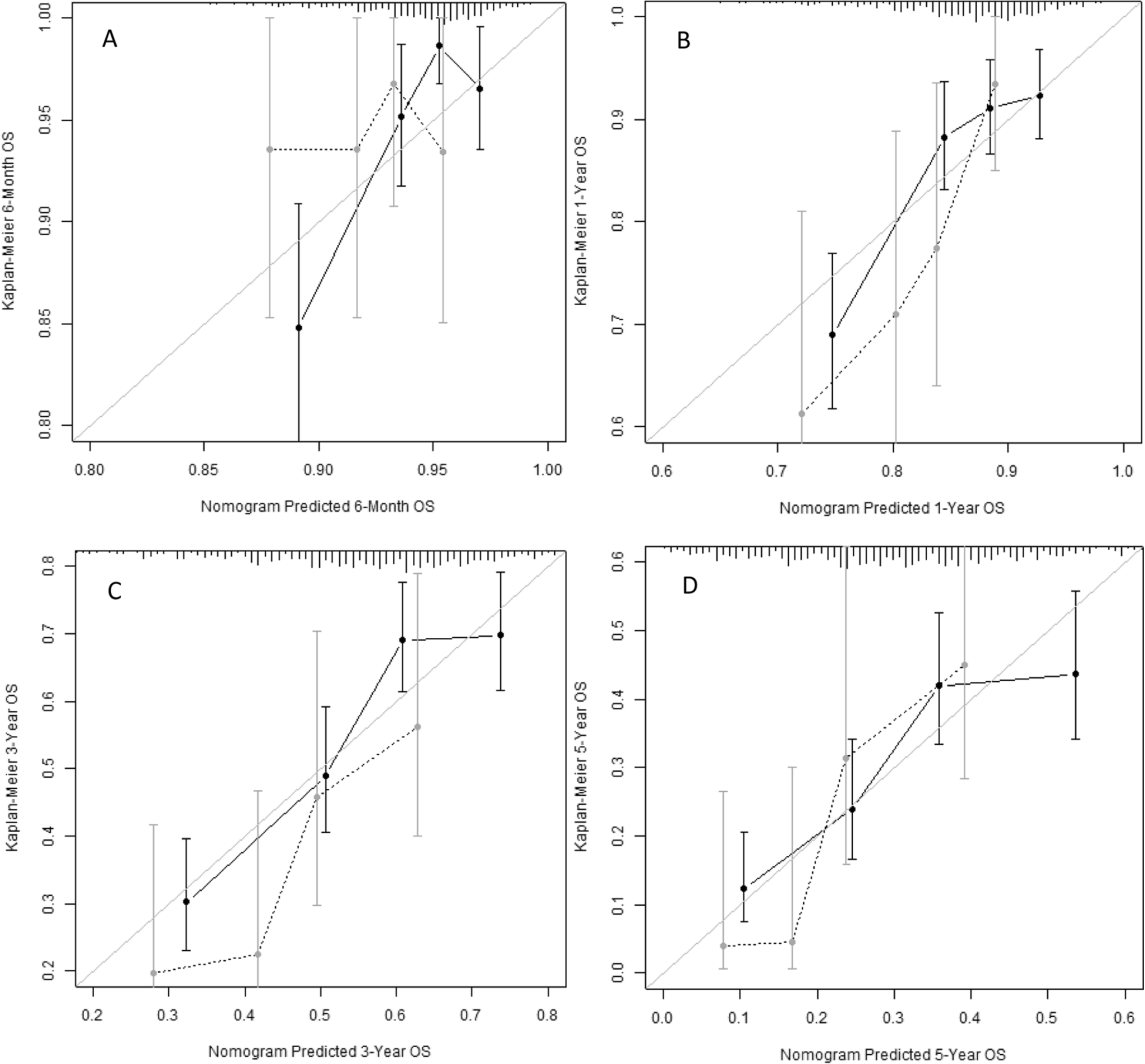
Calibration plots of Kaplan Meier vs nomogram predicted survival for the original patient group (black solid line) and the validation cohort (dotted grey line) for **a**) 6-month **b**) 1-year **c**) 3-year and **d**) 5-year overall survival. The error bars indicate the 95% confidence intervals. A plot along the 45-degree line would indicate perfect agreement between predicted and actual survival

The frequencies of prognostic factor selection in 1000 bootstrap samples are presented in Supplementary Table 1. KPS, age and tumor diameter were selected in 100% of samples, while operability was selected in 96%. The results of validating the model building procedure are presented in Supplementary Table 2. The C-index in the original sample was 0.66, and corrected for optimism through bootstrap sampling to 0.64. The optimism-corrected calibration slope was estimated at 0.766. Nevertheless, calibration plots demonstrated high correlation between observed and predicted probability of 6-month, 1-year, 3-year and 5-year OS (Fig. 4).

The model underlying the nomogram was used to predict OS of the patients from the external validation cohort. Its discriminative ability in this cohort as measured by C-index was assessed at 0.62, which is highly comparable with the results in the sample used to build the model. Fig. 4 presents calibration plots of the internal as well as the external validation.

## Discussion

Survival prediction at the individual patient level can facilitate informed treatment decisions for patients and clinicians. Here, we have developed a nomogram to predict OS, with moderate discriminatory ability (C-index 0.64), and good predictive accuracy based on calibration plots. The model displayed good external validity, with a C-index only slightly lower than that of the original cohort (C-index 0.62). Survival outcomes and baseline characteristics of the studied population are similar to those reported elsewhere [2, 18, 19], suggesting applicability of our model to other early NSCLC SBRT populations.

The prognostic importance of the six variables included in the final nomogram is corroborated by previous investigations. Age [10, 20, 21], sex [10, 20–23], performance status [3, 19, 24], operability [3, 25], tumor diameter [17, 22, 26, 27] and Charlson Comorbidity Index [3, 18, 20] have previously been reported as significant predictors of survival in the early NSCLC population. Interestingly, in the present sample smoking status was not significantly associated with survival, a finding reported previously [3, 18] although conflicting reports exists [19, 24].

Matsuo et al. [22] investigated prognostic factors in 101 patients with early stage lung cancer treated with SBRT and identified only male sex (HR 3.40, *p* = 0.004 on multivariate analysis) and tumor diameter (HR 1.60 per 10 mm increase, *p* = 0.013 on multivariate analysis) as adverse prognostic features for 3-year OS. The population was of atypically high performance status (94% World Health Organization performance status [WHO PS] 0–1) and operability (37% of patients) which may have accounted for the lack of association of age, performance status, and operability with survival. Of note, Matsuo et al. did not evaluate comorbidity as a potential predictor. Kopek et al. [18] did include Charlson Comorbidity score as a prognostic variable and found it to be a powerful predictor of survival: those with a CCI score of 6 or more had a median survival of only 11 months compared to 41 months in patients scoring 3 or less. T stage was also significant on multivariate analysis, and contrary to our findings, sex and performance status were not prognostic. Other variables lacking significance included histology and GOLD classification, consistent with our results.

The nomogram of the present study is one of only a few published for the early stage lung cancer population. A multi-institutional Chinese study developed a nomogram for OS in early stage lung cancer patients, however this was in the setting of resected disease [10]. Nevertheless, it shares similarities with the present nomogram, including incorporation of age, sex, and tumor size as prognostic variables. Although the C-index indicated good discriminatory ability at 0.71, the nomogram is not a useful predictive tool for patients undergoing lung SBRT for several reasons. It relies on surgical variables such as volume of blood loss and pathologic N stage. Additionally, comorbidity was not found to be significantly associated with survival and thus was not incorporated into the nomogram, but because it was coded in the model only as present or absent, if lacked the sensitivity of more established metrics such as CCI.

In the early lung SBRT population, Louie et al. also developed a nomogram for predicting OS, with a C-index similar to the present nomogram (0.66), however, it showed a lower degree of external validity (C-index 0.55 and 0.52 in two external validation cohorts) [19]. Our nomogram differs from that of Louie et al. in several key features. Only the nomogram presented here incorporates operability as a prognostic variable. As SBRT is increasingly applied to the operable setting, incorporating this important variable confers particular utility to our nomogram. Indeed, operability has previously been reported as an important prognostic factor [2, 3, 25]. Onishi et al. [25] reported 5-year overall survival for medically operable patients as 64.8%, compared to 35.0% in inoperable patients (*p* < 0.001). An additional distinction of the present nomogram is incorporation of KPS rather than WHO PS as a performance status metric. Performance status is perhaps the variable which most consistently appears as a prognostic factor for OS in early lung cancer, and with one of the greatest magnitudes of effect [3, 19, 24]. By utilizing KPS, which has a greater number of categories than WHO PS, our nomogram has greater discriminative ability for small differences in performance status which may significantly affect overall survival. Finally, our nomogram may also be used to predict 1-year and 3-year OS, and these shorter-term survival estimates may be particularly useful for treatment decision-making. The 5 year survival estimates generated by the nomogram, however, should be interpreted with caution, as the median follow up of the study was 24 months.

The nomogram’s short-term survival estimates warrant particular consideration. Very poor short-term prognosis may tip the balance in favor of a supportive care approach, sparing a patient the unnecessary inconvenience and potential cost of curative treatment. Due to the aggressive natural history of NSCLC, cancer-related morbidity and mortality can reasonably be anticipated within an approximately 1-year timeframe [28]. Hence, survival longer than 6 months likely warrants active treatment. Conversely, a low probability of 6-month survival may support a palliative approach. The present nomogram, however, generates a minimum 6-month survival estimate of 80%; adverse prognostic factors including advanced age and high CCI score did not confer a very low probability of short-term survival. This suggests that age and comorbidity burden are not sufficient to justify withholding curative-intent SBRT. It also highlights the need to better identify patient and disease factors predictive of early mortality [29]. Klement et al. [3] aimed to develop a model to predict early mortality in early-stage NSCLC patients undergoing SBRT, and similarly found that patients at high risk of early mortality could not be reliably identified: 6-month mortality was only 8.8% for the group of patients at highest risk, compared to 4.1% for those with the lowest risk.

Weaknesses of the study include its retrospective nature. Additionally, the external validation cohort consisted of patients treated also at our institution, while validation in a cohort from a distinct centre would better demonstrate generalizability of our nomogram. Finally, the majority of patients lacked a histopathologic diagnosis of lung cancer, such that this could not be included as a potential prognostic factor in the nomogram. Previous studies have suggested inferior outcomes for squamous cell carcinoma lung tumors treated with SBRT [30]. It is also possible that some benign tumors were included. However, the incidence of benign disease following surgery for Dutch patients with a clinical diagnosis of NSCLC is generally less than 5% [31], and SBRT outcomes in one study were no different with versus without pathologic confirmation of malignancy [31]. Molecular tumor markers were also not available. Strengths of the study include the relatively large patient population, homogenous treatment, and completeness of data and long-term follow-up. Calibration plots showed good agreement between nomogram-predicted and Kaplan-Meier-estimated survival, with excellent agreement for 3-year OS, suggesting high reliability of the nomogram. The nomogram was externally validated in a distinct patient population with central tumors, and despite difference from the original study population, the nomogram performed well in the external validation cohort. Development of a distinct nomogram for central lung tumors could be an avenue of future investigation, and could assess additional prognostic factors unique to central lung tumors such as potential tumor under-dosing in order to respect normal tissue tolerance. Future investigations may incorporate novel biomarkers and metabolomics signatures which are emerging as prognostic in the early NSCLC population [32].

## Conclusions

Here we present a validated a nomogram to predict OS in patients with early-stage NSCLC undergoing SBRT. The discriminatory ability is moderate and incorporation of emerging prognostic factors (for example molecular markers) may increase predictive ability for future models. Nevertheless, this prognostic tool may assist patients and clinicians in generating individual survival predictions.

## Supplementary information

**Additional file 1: Table S1.** Frequency of variable selection in 1000 bootstrap samples.

**Additional file 2: Table S2.** Results of internal validation of the model building procedure through 1000 bootstrap samples.

## Data Availability

The datasets used and/or analyzed during the current study are available from the corresponding author on reasonable request.

## Abbreviations

AIC: Akaike’s information criteria
BED: Biologically effective dose
CCI: Charlson comorbidity index score
CI: Confidence interval
CIRS: Cumulative illness rating score
d.f: Degrees of freedom
HR: Hazard ratio
KPS: Karnofsky performance status
LRT: Liklihood ratio test
NSCLC: Non-small cell lung cancer
OS: Overall survival
RCS: Restricted cubic splines
SBRT: Stereotactic body radiotherapy
