# Supplementary material for: Development and external validation of a nomogram to predict overall survival following stereotactic body radiotherapy for early-stage lung cancer
Source: Radiat Oncol. 2020 Apr 22;15:89. doi: 10.1186/s13014-020-01537-z (PMC7178957; doi:10.1186/s13014-020-01537-z)
Supplement: Supplementary file 1 — Additional file 1: Table S1. Frequency of variable selection in 1000 bootstrap samples. [file 13014_2020_1537_MOESM1_ESM.docx]

|  | Frequency of variable selection |
| --- | --- |
| KPS | 1000 |
| Age | 1000 |
| Tumor diameter | 1000 |
| Operability | 959 |
| CIRS | 552 |
| Sex | 531 |
| CCI | 448 |
| Previous malignancy | 461 |
| Previous lung cancer | 461 |
| Lower lobe location | 201 |
