# Supplementary material for: Development and external validation of a nomogram to predict overall survival following stereotactic body radiotherapy for early-stage lung cancer
Source: Radiat Oncol. 2020 Apr 22;15:89. doi: 10.1186/s13014-020-01537-z (PMC7178957; doi:10.1186/s13014-020-01537-z)
Supplement: Supplementary file 2 — Additional file 2: Table S2. Results of internal validation of the model building procedure through 1000 bootstrap samples. [file 13014_2020_1537_MOESM2_ESM.docx]

|  | Original sample | Training | Test | Optimism | Optimism-Corrected |
| --- | --- | --- | --- | --- | --- |
| C-index | 0.663 | 0.675 | 0.647 | 0.028 | 0.635 |
| Slope | 1.000 | 1.000 | 0.766 | 0.234 | 0.766 |
